# Efficacy of Hydrogen Peroxide Decontamination Against Food Pathogens on Chicken Wings and Removal by Catalase: In Vitro and Molecular Docking

**DOI:** 10.1021/acsomega.6c01860

**Published:** 2026-07-10

**Authors:** Mehmet Emin Aydemir, Dilara Erat, Mehmet Nuri Giraz, Adem Necip, Kasım Takim, Serap Kiliç Altun

**Affiliations:** † Department of Food Hygiene and Technology, Faculty of Veterinary Medicine, Harran University, 63300 Sanlıurfa, Türkiye; ‡ Department of Pharmacy Services, Health Services Vocational School, Harran University, 6330 Sanlıurfa, Turkey; § Department of Chemistry, Faculty of Arts and Sciences, Harran University, 63300 Sanlıurfa, Türkiye

## Abstract

This study aims to investigate the effects of decontaminating chicken wing meat with hydrogen peroxide (H_2_O_2_) at different concentrations and durations on the viability of *Salmonella* Typhimurium and *Listeria monocytogenes*, as well as the preservation of quality attributes in the chicken meat through the removal of H_2_O_2_ using the catalase enzyme. Additionally, the study aims to assess the impact of H_2_O_2_ on these pathogens through molecular docking analysis. To this end, contaminated chicken wings were submerged in different concentrations of H_2_O_2_ (5, 10, 25, 50, 100, and 250 ppm) for various durations (5, 15, 30, 45, 60, and 90 s). Following this, catalase enzyme solutions at different concentrations were used to remove H_2_O_2_. Approximately 3 log CFU/mL in rinsate samples reduction in *S.* Typhimurium was observed after 30 s of treatment with 250 ppm of H_2_O_2_, while *L. monocytogenes* counts decreased to below detectable levels after 90 s at 50 and 100 ppm and after 30 s at 250 ppm. Furthermore, it was observed that H_2_O_2_ was effectively removed by catalase enzyme treatment, and although statistically significant changes in color parameters were observed, no substantial adverse effect on overall color quality was evident. Molecular docking analysis indicated potential interactions of H_2_O_2_ with the selected target proteins under in silico conditions. The results obtained from this study suggest that such intervention strategies, applied toward the end of the processing chain before the packaging step, have potential as alternatives to other disinfectants like chlorine, which raise safety concerns.

## Graphical abstract

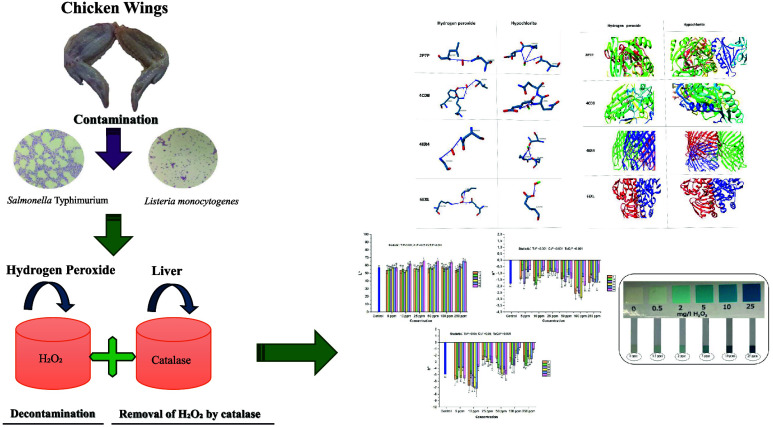

## 1 Introduction

The poultry meat industry represents a rapidly expanding sector within animal husbandry, capable of autonomously managing its production processes while significantly meeting the global demand for animal-derived protein. Due to its high bioavailability, rich protein content, adequate levels of essential amino acids, low fat content, and abundance of unsaturated fatty acids, poultry meat is considered a vital source of nutrition.^1,2^ In light of health concerns related to sedentary lifestyles and declining real income levels, there has been a shift in consumer preferences toward white meat, leading to an increased consumption trend. Consequently, as consumer incomes fall and issues persist within the production, supply, and marketing chains of red meat, poultry meat has emerged as a better cost-effective alternative for bridging the gap in animal protein supply.^3^ However, potential contamination by pathogenic and spoilage microorganisms in these products poses significant health risks and economic challenges for the poultry industry. In 2018, the European Union reported 94,203 confirmed cases of human salmonellosis and 2549 cases of listeriosis.^4^ Poultry meat and its derivatives are particularly vulnerable to contamination by pathogenic bacteria, such as *Salmonella* Typhimurium and *Listeria monocytogenes*, which can have adverse effects on public health.^5^

Poultry products are among the primary sources of foodborne Salmonella infections.^6,7^ Salmonella is responsible for approximately 47% of foodborne outbreaks globally, with 37% of these cases attributed to undercooked, contaminated poultry.^4,8^ The rising incidence of infections caused by this pathogen, coupled with the emergence of multiple antibiotic-resistant strains, has led to intensified control measures for Salmonella at various stages of animal production. Similarly, *L. monocytogenes* is a significant foodborne pathogen, responsible for fatalities in approximately 30% of infected individuals. Given its ubiquitous nature, failure to adhere to hygiene protocols can lead to contamination of meat and meat products.^9^ Ready-to-eat poultry products are frequently implicated in *Listeria* contamination.^10^ According to the Turkish Food Codex microbiological criteria, the presence of Salmonella in raw poultry meat and poultry mixtures, as well as the presence of *Salmonella* Spp and *L. monocytogenes* in heat-treated meat products, is prohibited.^11^

The role of slaughterhouses is pivotal in mitigating the risk of Salmonella contamination. As collection points for livestock from various ecological environments, slaughterhouses are prone to cross-contamination during the slaughter process, which can increase the microbial load. The effectiveness of control measures in slaughterhouses is therefore critical. Studies have shown that while the prevalence of Salmonella in live poultry is approximately 3–4%, this rate increases to 20–35% in the final product postslaughter.^12^

Contamination of chicken carcasses with bacteria during slaughter is inevitable. The slaughter process typically lasts 3–4 h, encompassing stages from scalding to packaging, during which bacterial contamination can occur. Consequently, decontamination strategies are essential for eliminating or reducing foodborne pathogens in poultry meat. Chlorine-based disinfectants are commonly employed for this purpose; however, their antimicrobial efficacy diminishes in the presence of organic matter due to the formation of chloramine. Furthermore, high concentrations of chlorine can result in undesirable odors and accelerate lipid oxidation in carcasses, which is why the recommended concentration range is restricted to 20–50 ppm.^13^ As a result, there is ongoing research into alternative disinfectants to chlorine.^14–16^

H_2_O_2_ is a potent bactericidal and bacteriostatic agent known for its rapid antimicrobial activity when used at appropriate concentrations.^17–20^ It is Generally Recognized as Safe (GRAS) by the U.S. Food and Drug Administration, and its application in food products has been approved.^21^ Recent reports also highlight its use in dairy products such as cheese.^22^ Furthermore, H_2_O_2_ has been demonstrated to be safe for use in meat and meat products when applied at proper concentrations and durations.^23^ However, the use of high doses of H_2_O_2_ in meat and meat products can result in residue issues, making the removal of H_2_O_2_ essential. Catalase, an antioxidant enzyme containing tetrameric iron porphyrin, catalyzes the reduction of H_2_O_2_ to water and oxygen, providing an effective strategy for eliminating H_2_O_2_ residues from meat.

Molecular docking has become an indispensable computational tool, primarily employed to elucidate protein–ligand interaction mechanisms. These interactions provide valuable insights into how molecular activity is enhanced as stable complexes are formed, offering significant information on the ways in which molecules interact with proteins to increase activity. The strength of interactions between molecules and proteins correlates directly with increased molecular activity.^24^ Therefore, molecular docking studies examining the antimicrobial effects of H_2_O_2_ against *S.* Typhimurium and *L. monocytogenes* provide crucial insights into its mechanism of action.

This study aims to investigate the effects of decontaminating chicken wing meat with H_2_O_2_ at varying concentrations and exposure times on the viability of *S.* Typhimurium and *L. monocytogenes*. Additionally, this study seeks to employ molecular docking analysis to further elucidate the antimicrobial mechanisms of H_2_O_2_. The study also aims to standardize methods for the efficient removal of H_2_O_2_ from chicken wing meat.

## 2 Materials and Methods

### 2.1 Preparation of H_2_O_2_ Concentrations

H_2_O_2_ solution was purchased from Merck (Darmstadt, Germany) for use in this study. The chemical properties of the H_2_O_2_ employed are as follows: a boiling point of 110 °C, molarity of 34.01 g/mol, density of 1.13 g/cm^3^, pH range between 2 and 4, vapor pressure of 20 hPa, and a concentration of 35% H_2_O_2_ suitable for use as an auxiliary substance. In this study, solutions were prepared freshly by serial dilution with sterile distilled water to obtain final concentrations of 5, 10, 25, 50, 100, and 250 ppm. All dilutions were calculated using the standard dilution equation (C_1_V_1_ = C_2_V_2_), and volumes were prepared in sterile conditions using calibrated volumetric equipment. The accuracy of prepared concentrations was verified by cross-checking dilution ratios and pH consistency of the solutions prior to application. All solutions were prepared immediately before use to ensure stability of H_2_O_2_ concentration.

### 2.2 Preparation of Catalase Enzyme

The catalase enzyme was prepared using two distinct methods. In the first method, catalase derived from bovine liver (Serva GMBH, Heidelberg, Germany) was purchased. This enzyme was diluted with distilled water at a 1:100 ratio to obtain a stock solution, which was then utilized in the study. In the second method, catalase was extracted from freshly slaughtered poultry liver. The liver was sterilized using 70% ethanol (Merck, Darmstadt, Germany), then homogenized using an IKA T25 Ultraturrax homogenizer (Staufen, Germany). The resulting homogenate was mixed with distilled water at a 1:10 ratio, shaken for 5 min, and filtered through filter paper. The supernatant obtained was collected and used as the stock solution for the study.

The activity of both commercial and poultry liver-derived catalase was determined spectrophotometrically according to the method described by Beers and Sizer (1952).^25^ The assay is based on monitoring the decrease in absorbance of H_2_O_2_ at 240 nm, using a molar extinction coefficient of 43.6 M^–1^ cm^–1^ for H_2_O_2_ calculations. One unit (U) of catalase activity was defined as the amount of enzyme required to decompose 1 μmol of H_2_O_2_ per minute at pH 7.0 and 25 °C. The specific activity levels for the commercial stock (0.002 U/mL) and the crude liver extract (0.48 U/mL) were standardized based on preliminary optimization trials, which identified these as the minimum effective concentrations required to reduce residual H_2_O_2_ to safe levels (<0.5 ppm) within a 60 s contact time.

### 2.3 Preparation of Chicken Wing Samples

Chicken wing samples, including the skin, were purchased from a local market on the day of the experiment. The samples were transported to the laboratory under cold chain conditions, and the experiments were conducted immediately upon arrival. To ensure the robustness of the findings, the experimental design was structured as follows: The experimental unit was defined as a single chicken wing piece (approximately 30 ± 5 g). The study was conducted using three independent biological replicates, where each replicate involved a completely new set of chicken wings purchased on different occasions and processed under identical conditions. Within each biological replicate, analytical replicates were performed by processing six individual wing pieces per treatment group. A total of 111 chicken wing pieces were utilized across the entire study (6 concentrations × 6 wings/group × 3 biological replicates +3 control samples). For randomization and group allocation, the chicken wing pieces were pooled together after arrival and then randomly assigned to the experimental groups using a random number table to eliminate potential bias related to initial microbial load or sample weight. A 1 mL sample of bacterial cocktail (6 log CFU/mL) was applied to the surface of each sample with spreading sticks and left to adhere for 10 min at room temperature. Inoculated chicken wings were randomly selected to form groups. The control group received no treatment (without H_2_O_2_). In the experimental groups, six different concentrations of H_2_O_2_ (5, 10, 25, 50, 100, and 250 ppm) were applied for various contact times (5, 15, 30, 45, 60, and 90 s). After decontamination, the chicken wings were immersed in catalase enzyme solutions to remove residual H_2_O_2_. All decontamination and H_2_O_2_ removal procedures were performed in sterile jars at 4 ± 1 °C. Each group of wings was treated in separate 500 mL jars. The selection of H_2_O_2_ concentrations and contact times was based on preliminary studies indicating that these conditions were sensorially acceptable.

### 2.4 Preparation of Pathogenic Bacterial Inoculum

*S.* Typhimurium (NCTC 12416, 74, and ATCC 14028) and *L. monocytogenes* (N 7144, RSKK 474 and 476, Refik Saydam National Public Health Institute, Turkey) reference strains were used for inoculating the chicken wing samples. Each bacterial strain was incubated in Tryptic Soy Broth at 37 °C for 18–24 h. After incubation, the bacterial pellet and supernatant were separated by centrifugation at 4000 rpm for 10 min. The pellet was washed in sterile isotonic NaCl solution (0.85% NaCl). The bacterial strains were then combined in separate tubes, and the bacterial count was adjusted to 10^8^/mL using the McFarland standard. The bacterial inoculum was diluted to achieve a contamination level of approximately 6 log on the chicken wing samples.

### 2.5 Decontamination of Chicken Wings

The contaminated chicken wing samples were allowed to adhere to the pathogens for 10 min before being immersed in H_2_O_2_ solutions at concentrations of 5, 10, 25, 50, 100, and 250 ppm for various contact durations (5, 15, 30, 45, 60, and 90 s). The contact time was precisely monitored using a laboratory timer. At the end of each treatment period, the samples were removed from the H_2_O_2_ solution and immediately immersed in catalase enzyme-containing solutions for H_2_O_2_ removal. The decontamination and H_2_O_2_ removal processes were performed in sterile jars with each group of wings treated in separate 500 mL jars containing 250 mL of H_2_O_2_ solution, ensuring complete immersion. The selection of H_2_O_2_ concentrations and contact times was based on preliminary studies indicating that these treatments were sensorially acceptable.

### 2.6 Analyses

#### 2.6.1 Analysis of H_2_O_2_ Removal and Quantification

To remove residual H_2_O_2_ from the chicken wings, catalase enzymes were employed. The catalase enzyme facilitates the breakdown of H_2_O_2_ into water and oxygen, as represented by the following reaction

$$H_{2}O_{2}+\mathrm{catalase}\to H_{2}O+O_{2}$$

The H_2_O_2_ removal processes were conducted in sterile jars at 4 ± 1 °C, with each group of wings treated separately in 500 mL jars containing 250 mL of catalase enzyme solution. The catalase concentration was optimized based on experimental results regarding time and concentration. The commercial catalase stock solution was used at 0.002 U/mL, effectively reducing H_2_O_2_ levels to below 0.5 ppm in 1 min. The catalase solution derived from poultry liver was used at a concentration of 0.48 U/mL, achieving similar H_2_O_2_ reduction. After treatment, residual H_2_O_2_ levels were measured using the MQuant Peroxide Test (Merck KGaA, Darmstadt, Germany), which is capable of measuring H_2_O_2_ levels up to 25 mg/L, in accordance with the manufacturer’s instructions.

#### 2.6.2 Microbiological Analyses

Following decontamination and H_2_O_2_ removal, the chicken wings were transferred to sterile Stomacher bags, and 100 mL of 0.1% peptone water (Merck, Darmstadt, Germany) was added. The samples were shaken by hand for 3 min, after which the wings were removed from the bags and the remaining rinse solution was used for microbiological analysis.^5^ Pathogen counts were determined using the spread plate method. *L. monocytogenes* was enumerated on Oxford agar (Merck, Darmstadt, Germany), and *S.* Typhimurium was enumerated on Xylose Lysine Deoxycholate (XLD) agar (Merck, Darmstadt, Germany). Plates were incubated at 35 °C for 24–48 h, and specific colonies (dark green-brown colonies with black-centered depressions for *L. monocytogenes* and black colonies for *S.* Typhimurium) were counted. Results were expressed as log10 CFU/mL.

#### 2.6.3 pH Analyses

The pH values of the samples were measured using a pH meter (EDT. GP 353), following the guidelines of AOAC (1990).^26^ The pH of the H_2_O_2_ solutions was measured by immersing the pH probe directly in the solutions.

#### 2.6.4 Color Analysis

The color of the chicken wing samples was assessed using a portable digital colorimeter (CS-10°, 8 mm, CHNSpec, Hangzhou, China). The results were expressed in terms of the L* (lightness/darkness), a* (redness/greenness), and b* (yellowness/blueness) parameters. Color measurements were taken at a minimum of three different locations on each sample at room temperature.

#### 2.6.5 Molecular Docking Analysis

The protein targets selected for molecular docking analysis include isocitrate lyase (PDB: 4KR4) and the DNA-binding protein (Dps) (PDB: 6EXL) from *S.* Typhimurium and listeriolysin O (LLO) (PDB: 4CDB) and internalin B (PDB: 2P7P) for *L. monocytogenes*. These proteins were selected due to their key roles in bacterial metabolism, the oxidative stress response, and virulence. Molecular docking studies were performed using the 3D structural data of target proteins, which were obtained from the Protein Data Bank (https://www.rcsb.org). Since no specific H_2_O_2_ ligand is present in the PDB, docking analysis was performed as an exploratory analysis by interpreting binding affinities based on the lowest binding energy and the frequency of hydrogen bond formation with conserved residues. Docking analyses were conducted with Chimera 1.17.3, AutoDock Vina, and Discovery Studio software. Ligand structures were drawn using ChemDraw 12.0, followed by energy minimization at the MM2 level using Chem3D Pro to obtain stable ligand configurations. The 3D protein structures were retrieved from the RCSB database, and the docking preparations involved the removal of steric clashes, addition of missing hydrogens, assignment of partial charges, construction of side chains, and filling of missing loops. Water molecules beyond the binding region were removed. It has been established that ligand-protein interactions are more stable and exhibit higher binding affinity at more negative binding energy values. The active site coordinates from the crystal structures were used as the basis for a redocking procedure. During the validation phase, predicted binding modes that achieved the lowest Root Mean Square Deviation (RMSD) values (typically <2.0 Å) were selected, and these refined positions were utilized for all subsequent analyses to ensure methodological accuracy. Protein–ligand interaction profiles were visualized in both 2D and 3D formats to analyze noncovalent interactions.^27^

#### 2.6.6 Statistical Analyses

The study was conducted with three independent replicates. Microbiological data were transformed into log 10 CFU/mL, and statistical analyses were performed using SPSS 24.0 for Windows (SPSS Inc., NY, USA). General Linear Models (GLM) were used to analyze the effects of H_2_O_2_ concentrations and contact times, with replicates as random effects. Multiple comparisons were made using Tukey’s test (*P* < 0.05), and a significance level of 0.05 was applied.

## 3 Results and Discussion

### 3.1 Microbiological Results of Chicken Wing Meat

The application of varying H_2_O_2_ concentrations and contact times significantly impacted the counts of *S.* Typhimurium and *L. monocytogenes* on the chicken wing samples. The detailed reductions in bacterial counts for both pathogens are presented in Table 1.

**Table 1:** Survival of *S.* Typhimurium and *L. monocytogenes* in H_2_O_2_ Solutions (Mean ± SE log_10_ CFU/mL)^a^

	time (second)	*S.* Typhimurium	log reduction	*L. monocytogenes*	log reduction
control^b^		5.59 ± 0.14^A^	0	5.30 ± 0.21^A^	0
5 ppm	5	5.23 ± 0.51^A^	0.36	5.60 ± 0.42^A^	–0.3
	15	5.17 ± 0.10^A^	0.42	5.00 ± 0.10^A^	0.3
	30	5.30 ± 0.49^A^	0.29	5.60 ± 0.42^A^	–0.3
	45	5.27 ± 0.28^A^	0.32	5.45 ± 0.63^A^	–0.15
	60	5.04 ± 0.49^A^	0.55	5.30 ± 0.10^A^	0
	90	5.08 ± 0.54^A^	0.51	5.34 ± 0.49^A^	–0.04
10 ppm	5	5.17 ± 0.2^A^	0.42	5.53 ± 0.33^A^	–0.23
	15	5.32 ± 0.32^A^	0.27	5.58 ± 0.15^A^	–0.28
	30	5.30 ± 0.41^A^	0.29	5.50 ± 0.28^A^	–0.20
	45	4.90 ± 0.56^A^	0.69	5.45 ± 0.21^A^	–0.15
	60	5.08 ± 0.54^A^	0.51	5.00 ± 0.10^A^	0.30
	90	4.93 ± 0.33^A^	0.66	5.00 ± 0.12^A^	0.30
25 ppm	5	4.99 ± 0.10^A^	0.60	5.70 ± 0.24^A^	–0.40
	15	5.13 ± 0.29^A^	0.46	5.62 ± 0.46^A^	–0.32
	30	5.00 ± 0.42^A^	0.59	5.69 ± 0.12^A^	–0.39
	45	4.83 ± 0.47^A^	0.76	5.23 ± 0.3^A^	0.07
	60	4.49 ± 0.91^A^	1.10	4.30 ± 0.10^B^	1.00
	90	4.42 ± 0.25^AB^	1.17	4.23 ± 0.10^B^	1.07
50 ppm	5	4.89 ± 0.10^A^	0.70	5.15 ± 0.21^A^	0.15
	15	4.98 ± 0.10^A^	0.61	4.65 ± 0.91^AB^	0.65
	30	4.20 ± 0.56^AB^	1.39	4.80 ± 0.95^AB^	0.50
	45	4.30 ± 0.13^AB^	1.29	5.00 ± 0.15^A^	0.30
	60	3.89 ± 0.10^AB^	1.70	2.7 ± 0.26^D^	2.60
	90	3.32 ± 0.88^B^	2.27	<1^E^	<4.30
100 ppm	5	4.58 ± 0.26^AB^	1.01	5.39 ± 0.12^A^	–0.09
	15	4.53 ± 0.10^AB^	1.06	4.65 ± 0.91^AB^	0.65
	30	4.54 ± 0.51^AB^	1.05	3.80 ± 0.28^BC^	1.50
	45	3.58 ± 0.15^B^	2.01	3.25 ± 0.49^C^	2.05
	60	3.31 ± 0.65^B^	2.28	2.10 ± 0.18^D^	3.20
	90	3.35 ± 0.91^B^	2.24	<1^E^	<4.30
250 ppm	5	3.95 ± 0.10^AB^	1.64	4.00 ± 0.10^BC^	1.30
	15	3.65 ± 0.14^B^	1.94	3.90 ± 0.14^BC^	1.40
	30	2.48 ± 0.12^C^	3.11	<1^E^	<4.30
	45	2.28 ± 0.26^C^	3.31	<1^E^	<4.30
	60	2.53 ± 0.33^C^	3.06	<1^E^	<4.30
	90	2.49 ± 0.28^C^	3.10	<1^E^	<4.30
Statistics	**T**	*P* < 0.001		*P* < 0.001	
	**C**	*P* < 0.05		*P* < 0.001	
	**TxC**	*P* < 0.001		*P* < 0.001	

a T: Time **C:** Concentration ^**A–E**^ **:** The mean values with different letters in the same column are significantly different (*P* < 0.05).

b The Control group in Table 1 received no treatment (without H_2_O_2_) and serves as the baseline for the initial bacterial population on the chicken wings before any intervention.

The most substantial reductions in *S.* Typhimurium counts were observed at all concentrations, particularly after 60 and 90 s of exposure. In the group treated with 250 ppm of H_2_O_2_, a reduction of approximately 3 log CFU/mL was observed from 30 s of exposure onward. These results indicate a time-dependent decrease in bacterial load, with higher concentrations and longer exposure times contributing to more significant reductions in *S.* Typhimurium.

Regarding *L. monocytogenes*, the counts were significantly reduced in all treatment groups, with the most notable decreases occurring in the 50 and 100 ppm concentrations after 90 s of exposure. Specifically, in these groups, the counts dropped to levels below 1 log CFU/mL. In the 250 ppm group, a similar reduction below 1 log was achieved after only 30 s of exposure. This suggests that *L. monocytogenes* is more susceptible to H_2_O_2_ treatment compared to *S.* Typhimurium, with a more rapid bactericidal effect observed at higher concentrations and shorter contact times.

These findings are consistent with previous studies that have demonstrated the effectiveness of H_2_O_2_ as a decontaminant against a range of foodborne pathogens.^17,20^ The time and concentration-dependent reduction in bacterial counts observed in this study emphasizes the critical role of both factors in optimizing decontamination protocols for poultry products.

The optimal pH range for *S.* Typhimurium is 6.5–7.5, although it can survive in environments with a pH as low as 4.5. In contrast, *L. monocytogenes* can tolerate pH levels between 4.0 and 5.5.^28^ However, exposure to acidic conditions, such as those created by H_2_O_2_, can adversely affect bacterial survival. Previous studies have shown that *L. monocytogenes* growth is inhibited under acidic conditions, as it tends to slow down and enter the stationary phase when exposed to lower pH levels.^29^

In the present study, the pH values of the H_2_O_2_ solutions used (as detailed in Table 2) may provide insight into the observed reductions in *S.* Typhimurium and *L. monocytogenes* counts on the chicken wing samples. Specifically, as the concentration of H_2_O_2_ increased, the pH of the solution decreased, which correlates with greater bacterial destruction. Moreover, longer exposure times further contributed to the reduction in bacterial counts, reinforcing the conclusion that both increased H_2_O_2_ concentrations and extended contact times facilitate more effective decontamination.

**Table 2:** pH Values of H_2_O_2_ Solutions (Mean ± SE)^a^

	pH value
Pure H_2_O_2_ ^b^	2.52 ± 0.35^C^
5 ppm	4.53 ± 0.19 ^A^
10 ppm	4.27 ± 0.24^AB^
25 ppm	4.05 ± 0.42^AB^
50 ppm	3.95 ± 0.43^AB^
100 ppm	3.65 ± 0.31^B^
250 ppm	3.56 ± 0.10^B^

a ^**A–D**^: The mean values with different letters in the same column are significantly different (*P* < 0.05).

b The pure H_2_O_2_ value represents the pH value of the pure H_2_O_2_ used in the study.

The reduction in *S.* Typhimurium and *L. monocytogenes* counts can be attributed to the antimicrobial properties of H_2_O_2_. H_2_O_2_ exerts its antibacterial effect by damaging bacterial DNA, proteins, lipids, and cell membranes, ultimately leading to microbial inactivation.^30^ Numerous studies investigating the application of H_2_O_2_ in various food matrices have demonstrated significant reductions in the population of key foodborne pathogens such as *S.* Typhimurium and *L. monocytogenes*, further substantiating its antibacterial efficacy.^5,9,22,31–35^

However, a comparison of the results from the present study with those of others reveals that the antibacterial effectiveness of H_2_O_2_ varies under different conditions. This variability is likely due to several factors, including the concentration of H_2_O_2_ used, treatment duration, temperature, microbial load, bacterial species, their resistance to H_2_O_2_, and the food matrix in which the bacteria are present.

In our study, at the highest concentration (250 ppm), while the count of *L. monocytogenes* decreased by less than 1 log, *S.* Typhimurium counts remained at approximately 2 log levels. This differential effect can be attributed to the resilience of the *S.* Typhimurium strains used, as well as their strong adhesion to the chicken meat. Indeed, incili et al. (2020)^5^ observed that *Salmonella* in marinated chicken pieces is less inactivated due to the protective effect of tightly adhering bacteria that shield them from the action of antimicrobial agents. Furthermore, a study supporting these findings indicated that as H_2_O_2_ concentration increased, the extracellular matrix of *L. monocytogenes* was weakened, membrane permeability was enhanced, and cell cytolysis was accelerated.^36^

The resistance observed in *S.* Typhimurium compared to *L. monocytogenes* raises important considerations regarding the prolonged use of disinfectants in food processing environments. In an industrial setting, repeated application of nonlethal H_2_O_2_ concentrations could theoretically facilitate the selection of bacterial strains with enhanced stress response mechanisms, such as increased production of endogenous catalase or specific DNA repair proteins (e.g., systems regulated by OxyR). Such adaptive resistance could lead to the emergence of strains less sensitive to oxidative processes over time.^37^ Although adaptive resistance was not investigated in the present study, this possibility should be considered in future studies evaluating the long-term industrial applications of H_2_O_2_-based decontamination strategies. In an industrial setting, to reduce the risk of selecting resistant phenotypes, intervention strategies should prioritize the rotational use of different disinfectant classes or the use of synergistic “barrier technology” approaches, ensuring that pathogen populations are not continuously exposed to a single selective pressure.

### 3.2 Results of H_2_O_2_ Removal in Chicken Wing Meat

To validate the methodology employed in this study, H_2_O_2_ solutions with concentrations ranging from 0 to 25 ppm were prepared, and trials were conducted. The results obtained from the test strips are presented in Figure 1, which also includes the color scale used for evaluating the H_2_O_2_ removal solutions in chicken wing meat.

**Figure 1:**
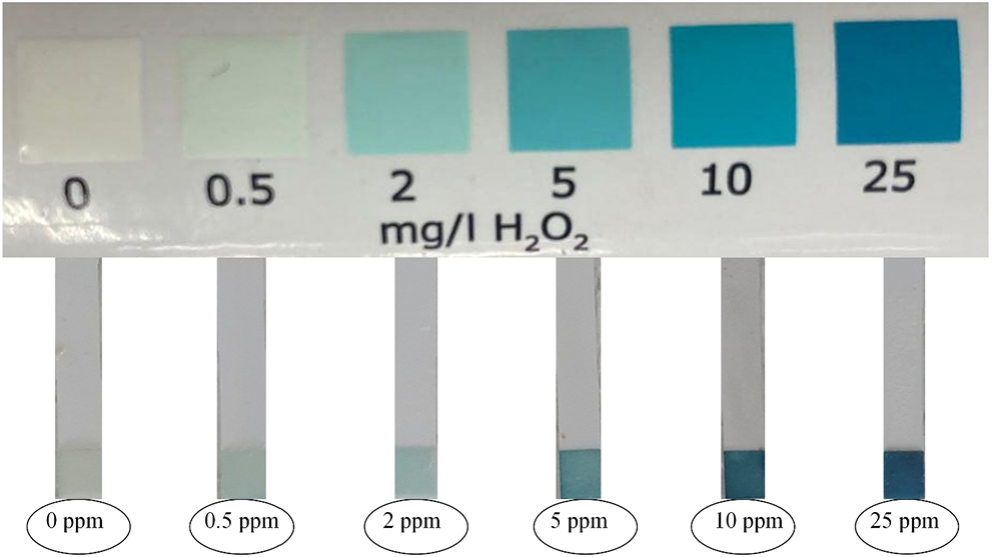
Peroxide test results in chicken wing removal water.

After the decontamination process, two distinct sources of catalase enzyme-commercial catalase and fresh poultry liver-were utilized to remove H_2_O_2_. It was determined that the specified amounts of catalase enzyme effectively removed H_2_O_2_ from chicken wings, and as shown on the test strips, residual H_2_O_2_ levels remained below the detection limit of the test method at all concentrations and decontamination times (Figure 1).

H_2_O_2_ is extensively utilized in the food industry across various countries. However, most industrial applications involving H_2_O_2_ require the rapid and efficient removal of this compound to ensure the production of high-quality end products, preserve nutritional value, and prevent the formation of toxic byproducts. Catalase is a well-established enzyme for the detoxification of H_2_O_2_. It has been employed in various forms and sourced from different organisms, with numerous studies reporting its efficacy in H_2_O_2_ removal.^38–40^ In the present study, catalase enzymes were found to effectively eliminate H_2_O_2_ from poultry meat. A similar methodology for measuring H_2_O_2_ accumulation on the surface of pig carcasses has been shown to yield reliable results using test strips.^41^ Thus, the method applied for H_2_O_2_ removal and residue detection in poultry meat processing can be considered efficient and applicable for industrial use.

In the present study, catalase enzymes were found to effectively eliminate H_2_O_2_ from poultry meat. The utilization of crude poultry liver extract was strategically chosen to propose a cost-effective and sustainable bioprocess by upcycling slaughterhouse byproducts into high-value functional processing aids. However, it is important to acknowledge certain limitations associated with the use of crude extracts, such as potential variations in protein concentration and enzymatic stability across different batches, compared to highly purified commercial enzymes. Despite these factors, the crude extract demonstrated comparable efficacy in H_2_O_2_ neutralization without inducing adverse effects on the meat’s physicochemical properties, justifying its potential for large-scale industrial applications where cost-efficiency is paramount. Thus, the method applied for H_2_O_2_ removal and residue detection in poultry meat processing can be considered efficient and applicable for industrial use.

### 3.3 Color Changes in Chicken Wing Meat

Color analysis results revealed that the L* value, which serves as a key indicator of poultry meat color quality, did not exhibit statistically significant changes when compared to the control group (Figure 2). However, a significant increase in L* values was noted in the 25 ppm group after 90 s of exposure, and in the 250 ppm group, a significant increase was observed after 60 s. Previous studies have reported L* values for chicken meat ranging from 40 (dark) to 79 (light),^42–47^ which align with the values observed in the current study. In a related study, the use of H_2_O_2_ as a decontaminant on poultry meat resulted in a white and swollen appearance of the meat, although no harmful effects were noted.^48^ The absence of similar effects in the present study can be attributed to differences in H_2_O_2_ concentrations, shorter application durations, and the prompt removal of H_2_O_2_ using catalase enzyme, which may have mitigated potential adverse effects on meat quality.

**Figure 2:**
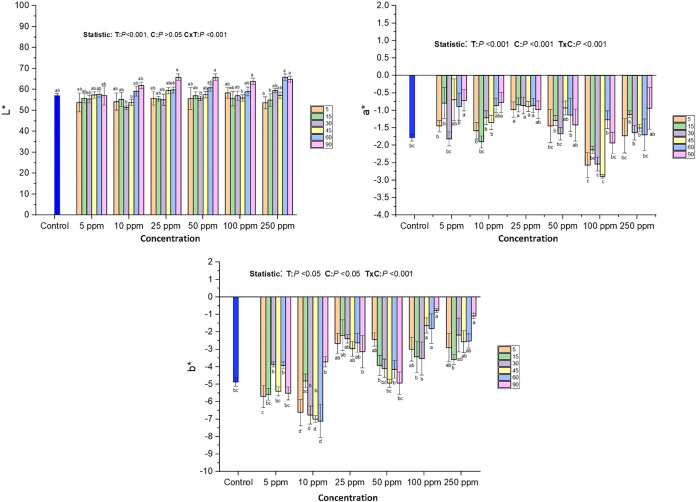
Color value changes in chicken wing meat at different concentrations of H_2_O_2_ (Mean ± SE). **T:** Time. **C:** concentration. ^**ab**^: Mean values with different letters are significantly different between groups (*P* < 0.05). L*: Whiteness/darkness. a*: Redness/greenness. b*: Yellowness/blueness.

Upon examining the color analysis results, it was observed that the application of H_2_O_2_ induced fluctuating changes in the a* and b* color parameters of chicken wing meat (Figure 2). The a* (redness/greenness) and b* (yellowness/blueness) values were found to be lower than those reported in previous studies.^47–49^ These differences can be attributed to the inherent variability in chicken meat or skin, the specific type of chicken used, the application method, exposure time, the color measurement device utilized, and the random positioning of color readings within each sample.

The observed fluctuations in the a* and b* color parameters can be further explained by changes in the pH of the meat. The procedures employed may have lowered the meat’s pH to the isoelectric point of myofibrillar proteins (approximately pH 5.0), which can lead to water loss, reduced moisture content, and alterations in meat color.^50^ Higher pH values have been associated with darker meat color,^51,52^ while pH reductions can result in lighter meat color.^53^ The findings of this study support the hypothesis that pH changes influence the color changes observed in chicken meat.

Additionally, the oxidative properties of H_2_O_2_ likely contributed to the observed lighter color of the meat. This whitening effect may explain the lower *a* and *b* values recorded. Similar effects have been documented for peracetic acid, another oxidative agent, which can alter the skin color of chicken depending on the concentration and exposure duration.^47^

Color analysis results revealed that while the application of H_2_O_2_ induced some fluctuations in color coordinates, although statistically significant changes in some color parameters were observed, these changes may have limited practical or sensory relevance. This is supported by the fact that the L* values remained within the range of naturally occurring variations reported for chicken meat.

The selection of H_2_O_2_ concentrations and treatment times is based on preliminary observations to ensure that the applications remain within a practically feasible range. While instrumental color analysis indicates that the overall appearance of chicken wings is preserved, it is important to note that no formal sensory panel evaluation was conducted. Future studies incorporating comprehensive sensory evaluations including odor, texture, and consumer taste profiles are necessary to fully validate the marketability of H_2_O_2_ disinfected poultry meat. Nevertheless, the current results provide a significant physicochemical and microbiological basis for evaluating the potential of H_2_O_2_ as a safe alternative to traditional disinfectants.

### 3.4 Molecular Docking Results

In the study, several parameters were calculated, each providing information about the different properties of the molecules. Among these parameters, the docking score is the first parameter that determines the activity of the molecules.

The docking score values of H_2_O_2_ and the positive control, hypochlorite, with the 4KR4, 6EXL, 4CDB, and 2P7P receptors are presented in Table 3. The affinity values for H_2_O_2_ ranged from −3.0 to −3.3 kcal/mol, while for hypochlorite, these values were between −2.1 and −2.6 kcal/mol. The best binding affinity was observed between H_2_O_2_ and the 4KR4 macromolecule, with a docking score of −3.3 kcal/mol. In the docking studies, H_2_O_2_ demonstrated higher affinity than the positive control across all macromolecules.

**Table 3:** Docking Score and H-bond Parameters of Hydrogen Peroxide and Hypochlorite against Proteins^a^

			hydrogen bonds				
		docking score (kcal/mol)	residue	AA	distance H–A	distance D–A	donor angle
Hydrogen peroxide	2P7P	–3.0	577E	ASN	2.13	3.11	170.93
			702F	LEU	2.63	3.08	108.10
	4CDB	–3.2	79A	VAL	2.13	3.07	159.49
			263A	GLN	2.11	3.08	166.84
			265A	TYR	2.45	3.21	138.19
	4KR4	–3.3	130A	GLY	2.38	3.15	134.73
			167A	ASN	2.39	3.08	126.27
	6EXL	–3.0	219A	LYS	2.55	3.15	116.79
			268B	GLN	2.84	3.23	105.00
			288B	ILE	2.38	3.09	128.80
Hypochlorite	2P7P	–2.4	522E	LYS	3.63	3.99	104.85
			523E	ASP	2.22	3.10	147.96
			523E	ASP	2.21	2.99	136.50
	4CDB	–2.6	111A	ARG	2.49	3.08	118.79
			111A	ARG	2.26	3.10	145.15
			112A	ASP	2.56	3.10	114.51
	4KR4	–2.1	68A	GLN	2.01	2.82	138.04
			69A	GLN	2.33	3.12	136.44
			749C	ASN	2.18	3.06	151.68
	6EXL	–2.4	273B	GLU	2.02	2.95	160.25

a **AA**: Amino acid.

It is known that proteins are inhibited due to their interactions with molecules. These interactions are generally chemical and involve hydrogen bonds. The greater the interaction between molecules and proteins, the higher the activity of the molecules. These interactions are typically of a chemical nature and include hydrogen bonds, polar and hydrophobic interactions, as well as π–π interactions. As these chemical interactions increase, the activity of the molecules also increases.^54^ The values calculated for each parameter and hydrogen bond are detailed in Table 3, which presents the docking scores and hydrogen bond parameters for H_2_O_2_ against these proteins. The three-dimensional molecular interactions of protein–ligand complexes are shown in Figures 3 and 4.

**Figure 3:**
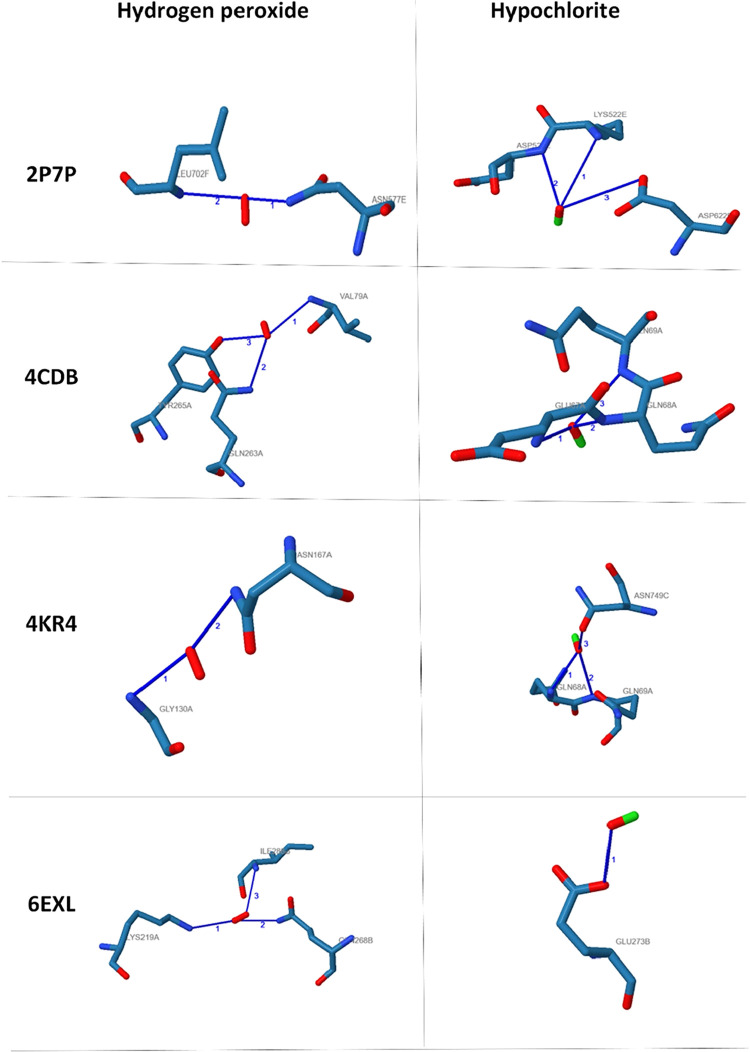
Two-dimensional (2D) representation of the interactions between hydrogen peroxide and hypochlorite and protein within the active site.

**Figure 4:**
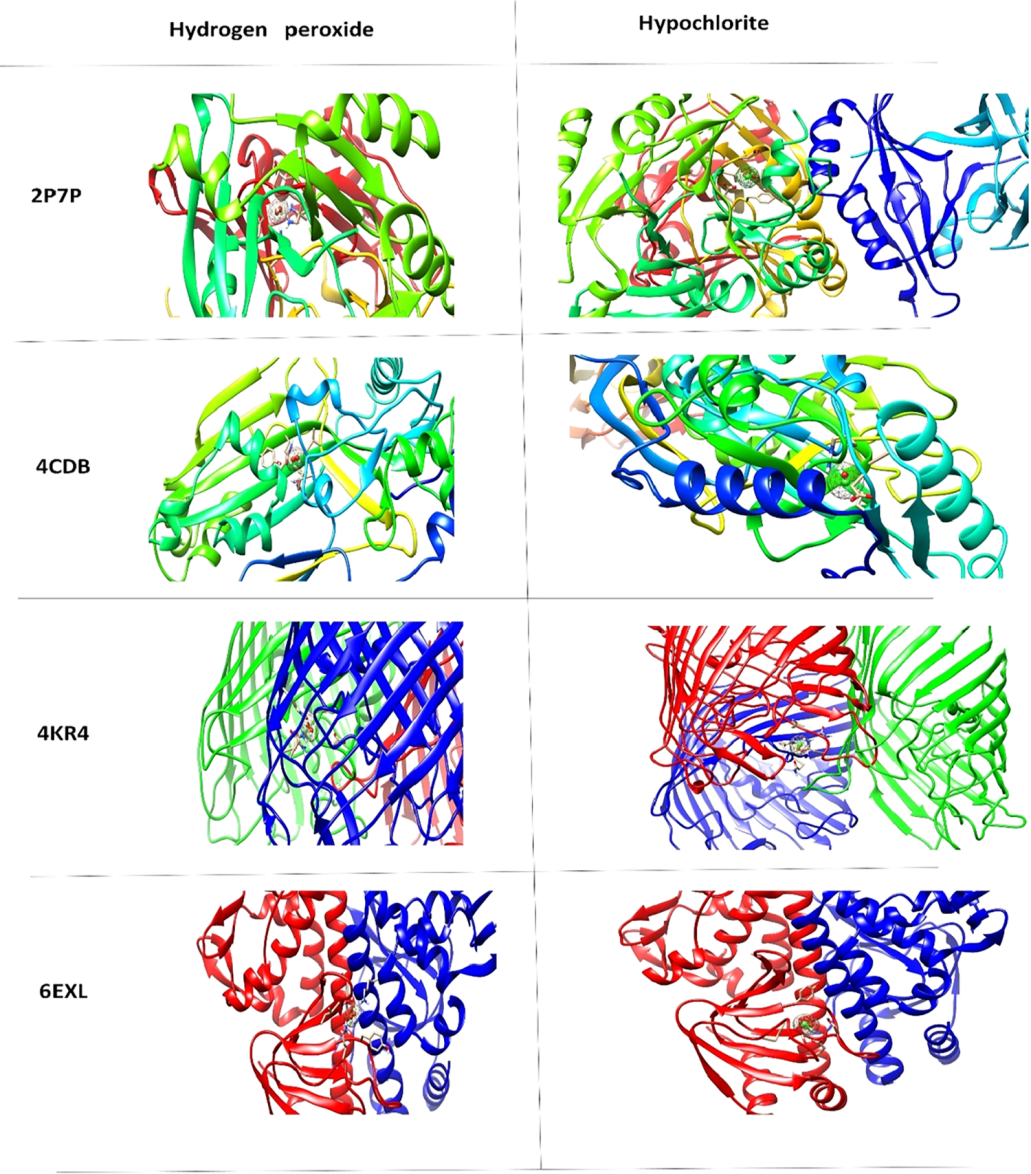
Three-dimensional (3D) representation of the interactions between the hydrogen peroxide and hypochlorite and the protein within the active site.

When the molecular docking results are evaluated, it was observed that H_2_O_2_ demonstrates greater effectiveness in terms of molecular interactions compared to sodium hypochlorite, which is commonly used for the decontamination of poultry meat against *S.* Typhimurium and *L. monocytogenes*. However, a study by Lineback et al. (2018)^55^ reported no significant difference in the antimicrobial effects between sodium hypochlorite and H_2_O_2_. This discrepancy can be attributed to the variability in disinfectant efficacy, which is influenced by factors such as the bacterial strain, pH differences of the disinfectants, and their distinct antimicrobial mechanisms.

In support of these findings, a study examining the effects of peracetic acid, a compound formed by the reaction of acetic acid and H_2_O_2_, and sodium hypochlorite on pathogens in poultry meat indicated that H_2_O_2_-based peracetic acid was more effective than sodium hypochlorite in reducing bacterial counts during storage periods.^56^ The results from the experimental study on the antimicrobial effects of H_2_O_2_ against *S.* Typhimurium and *L. monocytogenes* align with the outcomes observed in the molecular docking analysis, further supporting the efficacy of H_2_O_2_ as a potent decontaminant.

Molecular docking analysis has provided valuable theoretical insights into the interactions of H_2_O_2_ with selected target proteins under in silico conditions. However, these findings should be interpreted with caution, as the primary antimicrobial activity of H_2_O_2_ is associated with oxidative damage and reactive oxygen-mediated processes rather than specific ligand-like protein binding mechanisms.^57^ Therefore, the binding scores and hydrogen bond interactions observed in this study should be evaluated as supportive and exploratory data rather than definitive evidence of a specific ligand-binding mechanism. As demonstrated by Yildirim et al. (2025),^24^ molecular docking is a valuable computational tool for identifying potential interactions that can complement observed antimicrobial activity. These computational findings demonstrate that H_2_O_2_ has the potential to interact with functional proteins and potentially inhibit them, thereby complementing its primary mechanism of action through reactive chemical processes. This integrated approach provides a broader perspective on the multifaceted nature of H_2_O_2_ induced microbial inactivation.

This study evaluated the potential of H_2_O_2_ based interventions to enhance the safety of raw poultry meat and facilitate its removal via catalase enzyme. The results demonstrate that exposing chicken wing meat to H_2_O_2_ solutions at varying concentrations and durations effectively reduces *S.* Typhimurium and *L. monocytogenes*, with significant reductions in both pathogens observed as the concentration and exposure time increased. The catalase enzyme efficiently removed H_2_O_2_ across all concentrations and contact times, resulting in minimal changes to the quality characteristics of the meat. However, while statistically significant changes in some color parameters were observed, these changes may have limited practical or sensory relevance. Molecular docking analysis further revealed potential theoretical interactions between H_2_O_2_ and target proteins, providing exploratory insights that complement the observed in vitro pathogen inactivation.

In conclusion, this study demonstrated that H_2_O_2_ treatment provided significant reductions in *L. monocytogenes* and *S.* Typhimurium on chicken wing meat under specific concentrations and contact times. *L. monocytogenes* appeared to be more susceptible to H_2_O_2_ treatment than *S.* Typhimurium. Catalase treatment effectively reduced residual H_2_O_2_ levels to below the detection limit of the applied test method. Although statistically significant changes in some color parameters were observed, these changes appeared to have limited practical relevance to overall color quality. However, further research is needed to optimize these strategies and assess their broader applications in food safety, ensuring that food quality is preserved while minimizing risks in future poultry meat processing.
